# Working Memory and Executive Functions: Theoretical Advances

**DOI:** 10.5334/joc.424

**Published:** 2025-01-10

**Authors:** Sergio Morra, Steven J. Howard, Vanessa M. Loaiza

**Affiliations:** 1 Department of Education, Universitàdi Genova, Italy; 2 Early Start and School of Education, University of Wollongong, Australia; 3 School of Psychology, The University of Sheffield, United Kingdom

**Keywords:** working memory, executive functions, cognitive development

## Abstract

This editorial presents a special collection on working memory and executive functions. Six articles are presented and their contributions to current theoretical debates are briefly discussed.

Working memory and executive functions are constructs of crucial theoretical and practical importance because of their major role in higher cognition, intelligence, and cognitive control. However, they are also the grounds of hot scientific debate. Several theoretical issues are unresolved that also have important implications in applied contexts. When we launched the call for this special collection, we pointed to some issues that we felt to be in need of further discussion based on empirical research.

The first question that we raised was about the relation between working memory and executive functions: are they separate or related constructs, or is working memory an executive function itself? In their seminal article on executive functions, Miyake and colleagues identified inhibition, shifting and updating as three distinct but correlated basic executive functions, and suggested that “the ability to keep goal-related and other task-relevant information active in working memory during controlled processing could be the basis for the observed commonality among the three executive functions” (Miyake et al., 2000, pp. 88–89). However, in the field of developmental psychology, a majority of researchers follow the lead of Diamond (2013), who also theorized three executive functions, but listed them as inhibition, working memory, and cognitive flexibility. The concept that working memory is an executive function was reacted against by some researchers; for example, Oberauer (2009, p. 69) argued: “Nothing is to be gained for an understanding of working memory from using that concept.” Indeed, there has been some debate about the need of greater conceptual and terminological clarity, especially in developmental research (e.g., see Morra et al., 2018). However, none of the articles that we received for this special collection tackled explicitly our first question. Thus, in this editorial we can only remind the readers of the need to be clear in defining and using these constructs.

Our second question was about theoretical models in the field of working memory and/or executive functions, considering empirical support for any of them, and their possible comparison or integration. All six articles in this special collection have something to say in this regard, and we present them here under two headings – working memory and executive functions.

## Working memory

The first topic of the special issue concerned theoretical advances in working memory (Hautekiet et al., 2024; Johnson et al., 2024; Morra et al., 2024). Interestingly, all three papers concerned the use of attention in working memory, thus highlighting the growing importance of this field of research.

Johnson and colleagues (2024) investigated the important distinction between what they distinguish as mental attention versus automatic-perceptual attention in a sample of younger and older children from gifted versus mainstream classrooms. They employed two working memory tasks that respectively taxed the more effortful mental attention (i.e., n-back task) compared to the externally driven automatic-perceptual attention (i.e., self-ordered pointing task, SOPT) to test the hypothesis that gifted children would show an advantage on the former but not the latter task. Results showed that gifted children outperformed mainstream children on all tasks requiring mental attention, whereas no difference was observed for tasks primarily relying on automatic-perceptual attention. This finding provides insight into the measurement of attention in WM and its importance for developmental and individual differences in higher-order cognition more broadly.

In a similar vein, Morra and colleagues (2024) explored how the constraints of attention contribute to the well-known capacity limits of working memory, such that only a finite number of representations can be actively maintained and processed. Although historically this number has been proposed to be about seven units of information (Miller, 1956; Pascual-Leone & Johnson, 2011), others have instead suggested that it is likely closer to about four (Cowan, 2001; Luck & Vogel, 1997). Morra and colleagues (2024) thus designed a study to investigate the source of the different estimates of working memory capacity by varying the presentation rate of the stimuli in two canonical tasks from these respective perspectives: the compound stimuli visual information (CSVI) task and the visual array task (VAT). The results showed that the mean capacity estimates of the CSVI were stable around 6–7 units in young adults regardless of presentation rate, whereas the mean capacity estimate of the VAT was lower with brief presentation, but much greater under longer presentation rates. The authors concluded that the high inter-correlations between the capacity estimates between the two tasks indicate greater compatibility between the views than often conceded, such that discrepancies in capacity estimates may result from the different criteria adopted in different theoretical frameworks for identifying the relevant cognitive units.

Finally, Hautekiet and colleagues (2024) investigated a potential signature of the use of attention in working memory: as in perceptually driven exogenous attention (Posner & Cohen, 1984), people may be less likely to return their endogenous attention to a just-disengaged item compared to novel information (Johnson et al., 2013). Demonstrating this pattern of inhibition of return in both external and internal attention would indicate a close link between perception and working memory, but Hautekiet and colleagues (2024) argued that the original paradigm could be strengthened to more clearly identify the source of the pattern of slower responses to disengaged items. Indeed, when clarifying these potential methodological concerns, the results demonstrated that participants were slower to respond to a previously attended memory probe compared to a novel memory probe. This work helps to clarify how and when participants’ attention tends to either linger or switch between information in working memory, with further research necessary to determine whether these effects occur due to inhibitory versus reorienting attentional mechanisms.

In summary, the three papers on the first topic of this special issue provide important theoretical insights regarding the use of attention in working memory. The fact that all three of them focused on potential methodological issues that could contribute to differences between prior work in the literature highlights the fact that researchers across the field cannot take the parameters of their tasks and paradigms for granted when investigating fundamental research questions.

## Executive Functions

The second topic of the special issue considered theoretical advances in executive function more widely. The three articles taking an executive function focus targeted different issues, yet each is contentious in the executive function literature: the delineation, definition, and operationalisation of executive functions; how executive functions relate and changes in associations over time; and the under-representation of Majority World contexts in current executive function theorising and research.

Ionescu et al. (2024) ask whether cognitive flexibility is a domain-general or domain-specific capacity, given findings regarding set shifting (more narrowly) are often purposed for broader conclusions about cognitive flexibility. To evaluate this question, young adults from two countries completed a battery of assessments that required cognitive flexibility applied to contexts of language, mathematics, perception, or rule use. Findings indicated few and low correlation among the tasks. Relatedly, factor analysis indicated a 4-factor solution was optimal, largely separating by domain (except for a separation of response time and accuracy indices in assessment of cognitive flexibility in mathematics). While the authors note limitations of single indices of cognitive flexibility in each domain, this nevertheless serves as a provocation that cognitive flexibility may not be unitary, enabling flexible thinking and behaviour across domains. Possible alternatives are offered, such that ‘cognitive flexibility’ may instead be a product of other interacting mechanisms, or domain-bound duplications of the same core mechanism.

Menu et al. (2024) employed three parallel analytic approaches (i.e., SEM, network models, and, uniquely, latent variable network models) to evaluate previous findings on associations between executive functions and their differentiation over time. Longitudinal executive function data from young people – age 8 (at baseline) to age 14 (at endline) – were analysed. All approaches supported prior findings of executive-function-specific trajectories and increasing differentiation of executive functions with increasing age. Network models showed inhibition as most central to general executive processing in childhood, which was supplanted by updating in early adolescence. These findings provide more comprehensive and integrative insight into the structural organisation of executive functions from childhood to adolescence, offering insights to inter-executive-function interactions and timings that could be useful for intervention design. Despite the general convergence of results, a compelling case is made for careful consideration of analytic approach relative to the investigatory aims. Network analysis, for instance, may be a better suited approach to understanding relationships between executive function performances, indices and tasks–and, perhaps, characterising network-level executive function changes following intervention.

Finally, but no less fundamental, Cook et al. (2024) take up the issue that a majority of executive function theory and research is based on a minority of contexts in which the world’s children reside. They cite evidence that universality of those findings and the recommendations that arise therefrom must be evaluated, not assumed. In their own study, they investigate concurrent and longitudinal risk and protective factors of executive function in low-income South African children aged 3–5 years. Paralleling findings in other cultural contexts, attending formal early childhood education services was a significant predictor of executive function. Yet other predictors of executive function commonly found in Minority World settings were non-significant (e.g., caregiver education, household income) or in the opposite direction (i.e., more learning resources in the home was negatively associated with executive function). Novel predictors were also identified (i.e., greater diversity of caregivers was associated with higher executive function). Plausible explanations highlight unique contexts and conditions for this sample. The authors highlight the need for further research of this kind–but also qualitative, ethnographic methods to elucidate broader cultural and contextual influences on executive function and its development–and the need for these insights to be reconciled into a more complete, nuanced and global understanding of executive function.

While the diversity of topics across these papers precludes reconciled conclusions, each highlights important ongoing and future directions for executive function research if we are to achieve a more comprehensive, accurate, nuanced and global understanding of executive function.

## Conclusions

Although each article in this collection was quite specific in its research questions, they all offered valuable contributions relevant to the second question in our call – theoretical modelling. Menu et al. (2024), besides tackling important methodological issues, provide further support for the view of a developmental differentiation of executive functions during childhood (e.g., Karr et al., 2018) and the complexity of relations among executive function tasks. Ionescu et al. (2024) provide further evidence against the concept of a general dimension of cognitive flexibility, consistent with other, sparse previous research (e.g., Déak & Wiseheart, 2015); in this regard, we note that Cook et al. (2024) and Menu et al. (2024) use the term “cognitive flexibility” not with the very broad meaning questioned by Ionescu et al. (2024), but with the more specific meaning of “shifting” as defined by Miyake et al. (2000). Cook et al. (2024) offer a powerful reminder of the need to consider socio-cultural context (and in particular, the Majority World) when modelling executive function development. Hautekiet et al. (2024) clarify the working of the focus of attention, posited in several major models of working memory, and its relation to automatic inhibitory mechanisms. Johnson et al. (2024) provide further support for the distinction between effortful and automatic attention and their involvement in working memory tasks. Morra et al. (2024) suggest that Cowan’s (2001) and Pascual-Leone’s (1970) models of limited capacity are at least complementary, though not fully overlapping, because they refer to the same, limited attentional resource although they resort to different measurement systems counting only declarative or also procedural cognitive units.

A further question in the call for this special collection concerned practical implications. The work of Cook et al. (2024) has clear implications for assessment and intervention in disadvantaged contexts; also Johnson et al. (2024), Menu et al. (2024) and Morra et al. (2024) have at least indirect implications for testing practices.

Of course, six articles are just six more drops of water in the huge sea of working memory and executive function research. Hot scientific debates will probably remain hot for some time. As guest editors, we are happy with the valuable contributions offered by this special collection, and we expect further exciting developments from research in the forthcoming years.
